# Tsallis Entropy, Likelihood, and the Robust Seismic Inversion

**DOI:** 10.3390/e22040464

**Published:** 2020-04-19

**Authors:** Igo Pedro de Lima, Sérgio Luiz E. F. da Silva, Gilberto Corso, João M. de Araújo

**Affiliations:** 1 Programa de Pós-Graduação em Ciência e Engenharia de Petróleo - Universidade Federal do Rio Grande do Norte, Natal RN 59078-970, Brazil; igoivcds@hotmail.com (I.P.d.L.); gfcorso@gmail.com (G.C.); joaomedeiros@fisica.ufrn.br (J.M.d.A.); 2 Departamento de Física Teórica e Experimental, Universidade Federal do Rio Grande do Norte, Natal RN 59078-970, Brazil; 3 Departamento de Biofísica e Farmacologia, Universidade Federal do Rio Grande do Norte, Natal RN 59078-970, Brazil

**Keywords:** Tsallis entropy, maximum likelihood, *q*-Gaussian, inverse problems, seismic imaging

## Abstract

The nonextensive statistical mechanics proposed by Tsallis have been successfully used to model and analyze many complex phenomena. Here, we study the role of the generalized Tsallis statistics on the inverse problem theory. Most inverse problems are formulated as an optimisation problem that aims to estimate the physical parameters of a system from indirect and partial observations. In the conventional approach, the misfit function that is to be minimized is based on the least-squares distance between the observed data and the modelled data (residuals or errors), in which the residuals are assumed to follow a Gaussian distribution. However, in many real situations, the error is typically non-Gaussian, and therefore this technique tends to fail. This problem has motivated us to study misfit functions based on non-Gaussian statistics. In this work, we derive a misfit function based on the *q*-Gaussian distribution associated with the maximum entropy principle in the Tsallis formalism. We tested our method in a typical geophysical data inverse problem, called post-stack inversion (PSI), in which the physical parameters to be estimated are the Earth’s reflectivity. Our results show that the PSI based on Tsallis statistics outperforms the conventional PSI, especially in the non-Gaussian noisy-data case.

## 1. Introduction

Recently, Tsallis nonextensive entropy [1] was applied to describe and analyze many complex phenomena that the standard statistical mechanics seem inadequate to address [2,3,4]. The reason for the success of the Tsallis theory comes from the fact that the observational data are better described within the Tsallis framework [5]. In this scenario, the non-Gaussian distributions that arise from the Maximum Entropy Principle (MEP) [6,7] associated with Tsallis entropy [1,8] help to explain many characteristics of complex systems, such as long-range correlations [9] and the scale-free phenomena [10,11].

The Tsallis statistics are an extension of the standard statistical mechanics and they are based on the Boltzmann-Gibbs-Shannon (BGS) entropy measure. The successful applications of Tsallis entropy [12] motivates us to explore the mathematical tool behind the Tsallis framework. In this study, we included the Tsallis statistics in the inverse problems theory, which is an important field in applied physics [13], engineering [14], seismology [15], biomedical imaging [16], machine learning [17], and geophysics [18], among many others.

The essence of an inverse problem consists of obtaining information about physical parameters from indirect observations [18,19]. To achieve this task, the inverse problem is formulated as an optimisation problem that aims to minimize the difference between the modelled data and the observed data (residuals or errors), in which the usual misfit function is based on the least-squares distance of the residuals [19]. In addition, inverse problems are inherently ill-posed in the sense of Hadamard [20], meaning that the solution is not unique and a little perturbation in the observed data can severely impact the search for the global minimum of the misfit function [21].

In this paper, we explore a novel methodology in the geophysical data inverse problem using Tsallis entropy and a probabilistic maximum-likelihood approach. This methodology allows us to perform seismic data inversion to estimate the physical parameters of subsurface structures, known as post-stack inversion (PSI) [22]. The main objective of the PSI methodology is to estimate the reflectivity of subsurface structures by minimizing the difference between the observed seismic data and the theoretically modelled data using the misfit function [22,23]. We construct the misfit function by applying the probabilistic maximum-likelihood [19,24] method for the q-Gaussian probability distribution, which is obtained through the MEP for Tsallis entropy [8,25,26]. From a statistical viewpoint, this is equivalent to assuming that the residuals obey the q-generalized Gauss’ error law [27]. The results show that the PSI based on Tsallis statistics is a powerful tool for the reconstruction of seismic images, especially in situations where the data is noisy. Our proposal outperforms the conventional PSI approach because the noise present in the data is seldom Gaussian [28].

In the next section, we briefly review the conventional PSI formulation. In Section 3, we review the Tsallis framework and derive the misfit function associated with the nonextensive entropy, as proposed in the Tsallis work [1]. This section is the core of this work and, therefore, it is dedicated to PSI formulation on the Tsallis framework. In Section 4, we describe the parameters that we used in the numerical simulations to test our methodology to a geophysical problem from oil reservoir imaging. In addition, we compare the conventional Gaussian-oriented PSI with the PSI based on q-statistics. Finally, in Section 5, we give our final remarks and we place this manuscript in a broader context.

## 2. Conventional PSI Formulation

The PSI is used to quantitatively infer rock-properties. In particular, it describes the subsurface structures from the reflection data (or post-stack data) recorded in a seismic survey [22]. The typical seismic experiment consists of an explosive source and a set of mechanical sensors that capture the waves reflected from the geological structures. Using a conventional approach, the PSI is formulated as a least-squares optimisation problem [22,23], whose main goal is to minimize the difference between the observed data ($d^{obs}$) and the modelled data ($d^{mod}$) using the misfit function:

(1) $$\underset{r}{\min}\;\phi_{G}\left(r\right):=\frac{1}{2}\sum_{i=1}^{n}{\left(d_{i}^{mod}\left(t,r\right)-d_{i}^{obs}\left(t\right)\right)}^{T}\left(d_{i}^{mod}\left(t,r\right)-d_{i}^{obs}\left(t\right)\right)$$

In this equation, r represents the reflectivity of the medium under study (model parameters) and *t* denotes the time. In addition, the superscript *T* refers to the transpose operator and *n* represents the size of the total reflectivity series recorded in the seismic survey.

The modelled data relies on the convolution between the seismic source, s(t), and the medium reflectivity series, r(t), given by [29]:

(2) $$d^{mod}\left(t,r\right)=s\left(t\right)*r\left(t\right)=Gr\left(t\right)$$

where * represents the convolution operator and *G* represents a kernel matrix computed from the seismic source, in which its action on any vector r(t) will result in the convolution between s(t) and r(t).

The least-squares PSI (hereafter conventional PSI) determines the *maximum a posteriori* solution that can be used to estimate the model parameters. From a probabilistic maximum-likelihood viewpoint, the errors are assumed to be independent and identically distributed by a Gaussian probability distribution [18,19,24]. Consequently, the minimization of $\phi_{G}\left(r\right)$ is equivalent to the maximization of the standard Gaussian likelihood, given by:

(3) $$L_{G}\left(r\right)\propto \exp\left[-\frac{1}{2}\sum_{i=1}^{n}{\left(d_{i}^{mod}\left(t,r\right)-d_{i}^{obs}\left(t\right)\right)}^{T}\left(d_{i}^{mod}\left(t,r\right)-d_{i}^{obs}\left(t\right)\right)\right]$$

in which the *maximum a posteriori* of **r** is obtained by minimizing the negative log of $L_{G}\left(r\right)$.

It is well-known that the MEP [6,7] for the BGS entropy

(4) $$S\left(p(x)\right)=-\int p\left(x\right)\;\ln\left(p(x)\right)dx$$

under the constraints

(5) ∫p(x)dx=1

and

(6) $$\int x^{2}p\left(x\right)dx=1.$$

straightforwardly yields a standard Gaussian distribution, where p(x) is a probability density function. The constraint (5) is the normalization condition, while the constraint (6) restricts the second moment to unity. In summary, the conventional PSI maximizes the BGS entropy under the constraints (5) and (6).

## 3. Tsallis Framework and Seismic Inversion

Constantino Tsallis postulated a generalization of the BGS entropy [1] that describes non-equilibrium states and also describes the equilibrium thermostatistics [30]. Formally, the Tsallis framework (or *q*-framework) is based on the *q*-generalized logarithm function [1,8]

(7) $$\ln_{q}\left(x\right)=\frac{x^{1-q}-1}{1-q}$$

and its inverse function, the *q*-generalized exponential:

(8) $$\exp_{q}\left(x\right)={\left[1+(1-q)x\right]}_{+}^{\frac{1}{1-q}}$$

with

(9) $$\ln_{q}\left(\exp_{q}\left(x\right)\right)=\exp_{q}\left(\ln_{q}\left(x\right)\right)=x$$

where q∈R is the nonextensive parameter of the Tsallis theoretical framework and ${\left[a\right]}_{+}=\max\left\{0,a\right\}$. In the q→1 case, the expressions in (7) and (8) reduce to the usual exponential and logarithmic functions, respectively. For the continuous case, the *q*-entropy associated with the *q*-framework is given by:

(10) $$S_{q}=-k_{B}\int p^{q}\left(x\right)\;\ln_{q}(p\left(x\right))dx$$

where $k_{B}$ is the Boltzmann constant. In the limit q→1, the *q*-entropy in (10) recovers BGS entropy [1]

(11) $$S_{q\to 1}=\underset{q\to 1}{\lim}\;-k_{B}\int p^{q}\left(x\right)\;\ln_{q}(p\left(x\right))dx=-k_{B}\int p\left(x\right)\;\ln(p\left(x\right))dx$$

Furthermore, Tsallis entropy (10) is usually written as [1]:

(12) $$S_{q}\left(p(x)\right)\equiv \frac{k_{B}}{q-1}\left(1-\int p^{q}\left(x\right)dx\right)$$

which is the mathematical definition that we will use in this paper.

### 3.1. Maximum Tsallis Entropy and the *q*-Gaussian Distribution

The MEP [6,7] for Tsallis entropy (12) under the constrains of normalization (5) and the *q*-normalized expectation

(13) $$\sigma_{q}=\frac{\int x^{2}p^{q}\left(x\right)dx}{\int p^{q}\left(x\right)dx}$$

is associated with the following functional entropy to be maximized:

(14) $$S\left(p\left(x\right),\alpha_{1},\alpha_{2}\right)=S_{q}\left(p\left(x\right)\right)-\alpha_{1}\left(\int p(x)dx-1\right)-\alpha_{2}\left(\int x^{2}p^{q}\left(x\right)dx-\sigma_{q}\int p^{q}\left(x\right)dx\right)$$

where $\alpha_{1}$ and $\alpha_{2}$ are Lagrange multipliers.

The optimisation of Equation (14) consists of calculating the functional entropy saddle point (stationary point), $\frac{\delta S}{\delta p(x)}=0$:

(15) $$\int \delta p\left(x\right)\left[\frac{q\;k_{B}}{1-q}p^{q-1}\left(x\right)-\alpha_{1}-q\;\alpha_{2}\left(x^{2}p^{q-1}\left(x\right)-\sigma_{q}p^{q-1}\left(x\right)\right)\right]dx=0$$

To satisfy Equation (15) the following equation should be satisfied:

(16) $$\frac{q\;k_{B}}{1-q}p^{q-1}\left(x\right)-\alpha_{1}-q\;\alpha_{2}\left(x^{2}p^{q-1}\left(x\right)-\sigma_{q}p^{q-1}\left(x\right)\right)=0$$

which straightforwardly yields the *q*-probability function:

(17) $$p\left(x\right)={\left[\left(\frac{q}{\alpha_{1}}\right)\left(k_{B}-\left(1-q\right)\alpha_{2}\left(x^{2}-\sigma_{q}\right)\right)\right]}^{\frac{1}{1-q}}$$

To rewrite the probability function in (17) using *q*-generalized functions, consider the expression

(18) $$p\left(x\right)={\left[1+\left(1-q\right)A_{q}\right]}^{\frac{1}{1-q}}{\left[1+\left(1-q\right)\left(-B_{q}\right)x^{2}\right]}^{\frac{1}{1-q}}$$

where $A_{q}$ and $B_{q}$ depend on *q* and the Lagrange multipliers $\alpha_{1}$ and $\alpha_{2}$. After some algebra, we derive the following expression for the q-probability distribution:

(19) $$p\left(x\right)=\exp_{q}\left(A_{q}\right)\;\exp_{q}\left(-B_{q}\;x^{2}\right)$$

with

(20) $$A_{q}=\frac{q}{\alpha_{1}\left(1-q\right)}\left[\frac{k_{B}}{1-q}+\alpha_{2}\sigma_{q}-\frac{\alpha_{1}}{q}\right]\;\mathrm{and}\;B_{q}=\frac{\alpha_{2}}{k_{B}+\left(1-q\right)\alpha_{2}\;\sigma_{q}}$$

In this context, Equation (19) corresponds to the *q*-Gaussian probability distribution, which is used in the geophysical inversion in this manuscript. We notice that no *q*-Gaussian exists in the q≥3 case because it does not satisfy the normalization condition, Equation (5).

### 3.2. The *q*-misfit Function

Assuming that all residual data ($x=x_{1},x_{2},\ldots ,x_{n}$) are independent and follow a *q*-Gaussian distribution (20), the *q*-misfit function is obtained by the log-likelihood (we take the logarithm for convenience):

(21) $$-\ln\left(L_{q}\left(x\right)\right)=-\ln\left(\prod_{i=1}^{n}p_{q}\left(x_{i}\right)\right)=-\ln\left(\prod_{i=1}^{n}\exp_{q}\left(A_{q}\right)\;\exp_{q}\left(-B_{q}\;x_{i}^{2}\right)\right)$$

This formula can be written as:

(22) $$-\ln\left(L_{q}\left(x\right)\right)=-\ln\left[\exp_{q}\left(A_{q}\right)\right]-\frac{1}{1-q}\;\sum_{i=1}^{n}\ln{\left[1+\left(1-q\right)\left(-B_{q}\right)\;x_{i}^{2}\right]}_{+}$$

We notice that minimize Equation (22) is equivalent to minimizing the function:

(23) $$\phi_{q}\left(x\right)=\frac{1}{q-1}\;\sum_{i=1}^{n}\ln{\left[1+\left(q-1\right)B_{q}\;x_{i}^{2}\right]}_{+}$$

which in our geophysical inversion problem is formulated as the following optimisation problem:

(24) $$\underset{r}{\min}\;\phi_{q}\left(r\right):=\frac{1}{q-1}\;\sum_{i=1}^{n}\ln{\left[1+\left(q-1\right)B_{q}\;{\left(Gr_{i}\left(t\right)-d_{i}^{obs}\left(t\right)\right)}^{T}\left(Gr_{i}\left(t\right)-d_{i}^{obs}\left(t\right)\right)\right]}_{+}$$

In addition, we consider that $B_{q}=1/\left(3-q\right)$, as proposed in [25] for 1<q<3. We notice that the conventional misfit function is recovered from Equation (24) for the limit q→1. Using the L’Hôpital’s rule:

$$\lim_{q\to 1}\phi_{q}\left(r\right)=\lim_{q\to 1}\sum_{i=1}^{n}\frac{\frac{\partial }{\partial q}\left\{ln{\left[1+\left(\frac{q-1}{3-q}\right){\left(Gr_{i}\left(t\right)-d_{i}^{obs}\left(t\right)\right)}^{T}\left(Gr_{i}\left(t\right)-d_{i}^{obs}\left(t\right)\right)\right]}_{+}\right\}}{\frac{\partial (q-1)}{\partial q}}$$

$$\lim_{q\to 1}\phi_{q}\left(r\right)=\lim_{q\to 1}\sum_{i=1}^{n}\frac{{\left(\frac{1}{3-q}\right)}^{2}{\left(Gr_{i}\left(t\right)-d_{i}^{obs}\left(t\right)\right)}^{T}\left(Gr_{i}\left(t\right)-d_{i}^{obs}\left(t\right)\right)}{\;{\left[1+\left(q-1\right)B_{q}{\left(Gr_{i}\left(t\right)-d_{i}^{obs}\left(t\right)\right)}^{T}\left(Gr_{i}\left(t\right)-d_{i}^{obs}\left(t\right)\right)\right]}_{+}\;}$$

(25) $$\lim_{q\to 1}\phi_{q}\left(r\right)=\frac{1}{2}\sum_{i=1}^{n}{\left(Gr_{i}\left(t\right)-d_{i}^{obs}\left(t\right)\right)}^{T}\left(Gr_{i}\left(t\right)-d_{i}^{obs}\left(t\right)\right)=\phi_{G}\left(r\right)$$

*quod erat demonstrandum.*

Therefore, the optimisation problem formulated in (24) becomes:

(26) $$\underset{r}{\min}\;\phi_{q}\left(r\right):=\frac{1}{q-1}\;\sum_{i=1}^{n}\ln{\left[1+\left(\frac{q-1}{3-q}\right)\;{\left(Gr_{i}\left(t\right)-d_{i}^{obs}\left(t\right)\right)}^{T}\left(Gr_{i}\left(t\right)-d_{i}^{obs}\left(t\right)\right)\right]}_{+}$$

We call the function $\phi_{q}\left(r\right)$ a *q*-misfit function and we call *q*-PSI the optimisation problem employing the *q*-misfit function. It is worth noting that the term in brackets is always positive in the 1<q<3 case. However, for q<1, $\phi_{q}\left(r\right)$ is given by Equation (26) if $|Gr_{i}\left(t\right)-d_{i}^{obs}{\left(t\right)|<\left[\left(3-q\right)/\left(1-q\right)\right]}^{1/2}$ [25] and zero otherwise. To simplify the notation, henceforth operation ${\left[a\right]}_{+}$ will be implicit in the equations.

### 3.3. PSI as a Local Optimisation Problem

As mentioned earlier, the PSI is usually solved using local optimisation methods. Starting from a reflectivity series $r_{0}$ (initial model), the optimisation problem is solved iteratively by updating the model according to:

(27) $$r_{k+1}=r_{k}-\alpha_{k}H^{-1}\nabla_{r}\phi \left(r_{k}\right)$$

where $\alpha_{k}$ is the steplength [31] of the *k*-th iteration, H is the Hessian matrix, and $\nabla_{r}\phi \left(r_{k}\right)=\partial \phi \left(r_{k}\right)/\partial r$ is the gradient of the misfit function $\phi (r_{k})$. Therefore, in addition to the misfit function, we need to determine their corresponding gradient and Hessian.

In the *q*-PSI case, the gradient is given by:

(28) $$\nabla_{r}\phi_{q}\left(r\right)=\sum_{i=1}^{n}G^{T}\left[\frac{2\;\left(Gr_{i}\left(t\right)-d_{i}^{obs}\left(t\right)\right)}{3-q+\left(q-1\right)\;{\left(Gr_{i}\left(t\right)-d_{i}^{obs}\left(t\right)\right)}^{T}\left(Gr_{i}\left(t\right)-d_{i}^{obs}\left(t\right)\right)}\right]$$

For comparison, the gradient of the conventional misfit function is given by:

(29) $$\nabla_{r}\phi_{G}\left(r\right)=\sum_{i=1}^{n}G^{T}\left(Gr_{i}\left(t\right)-d_{i}^{obs}\left(t\right)\right)$$

wherein it is easy to see that at the limit q→1, the q-misfit function gradient—Equation (28)—becomes the conventional misfit gradient function—Equation (29).

By comparing Equations (28) and (29), we can see that the gradient of the q-misfit function is weighted by the factor $2/[3-q+\left(q-1\right){\left(Gr_{i}\left(t\right)-d_{i}^{obs}\left(t\right)\right)}^{T}\left(Gr_{i}\left(t\right)-d_{i}^{obs}\left(t\right)\right)]$. Consequently, the large residual data are dampened in the model update process, Equation (27), which makes the *q*-PSI less sensitive to large errors than the conventional PSI approach.

In our numerical study, because of the fast convergence, we perform the optimisation with the quasi-Newton method *limited*-*memory* Broyden-Fletcher-Goldfarb-Shanno (*l*-BFGS) [31], in which the inverse of the Hessian is obtained through an approximation computed from previous gradients [32]. Thus, we do not explicitly calculate the second derivative of the misfit function, which saves a lot of memory and reduces the computational cost. This trick is valuable because geophysics problems typically have a large number of variables (number of model parameters).

## 4. Numerical Results

**Figure 1:**
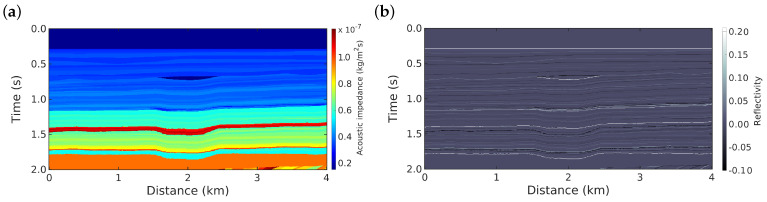
(**a**) The portion of the Marmousi2 acoustic impedance model in the depth-time domain. (**b**) Reflectivity model (true model), which is extracted from the impedance model in Figure 1a using Equation (30).

We illustrate our method using a portion of the Marmousi2 model, which is based on the geology of the Kwanza Basin region (Angola) [33,34]. The acoustic impedance distribution of this model is shown in Figure 1a. The impedance model consists of 2000 and 400 grid cells in the vertical and horizontal directions, respectively (800,470 total grid points). From this impedance model, we obtained the reflectivity model that will be used as the true model in this study, which is depicted in Figure 1b. The mathematical relationship between acoustic impedance (*Z*) and reflectivity (*r*) is given by [29,35]:

(30) $$r\left(t\right)=\frac{1}{2}\frac{d}{dt}\left[\ln\left(Z(t)\right)\right]$$

A Ricker wavelet [36] is considered to be the source signature, which is defined by:

(31) $$s\left(t\right)=\left(1-2\pi^{2}\nu_{p}^{2}t^{2}\right)\exp\left(-\pi^{2}\nu_{p}^{2}t^{2}\right)$$

where $\nu_{p}$ is the most energetic frequency. In this study, we considered $\nu_{p}=55$ Hz.

**Figure 2:**
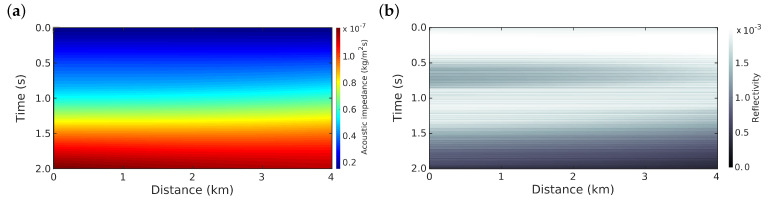
(**a**) Initial acoustic impedance model in the depth-time domain, and its (**b**) reflectivity model (Initial model).

For the optimisation solver, we use the *l*-BFGS algorithm [32] to minimize the misfit function for both methods, in which the stop criteria is a tolerance ϵ in the gradient norm or if the step length does not satisfy the Wolfe conditions [31]. The gradient norm is $\left|\right|\nabla_{r}\phi \left(r\right)\left|\right|<\epsilon =10^{-12}$. If one of these criteria is satisfied, then the inversion process stops. Figure 2b shows the initial model that was used for all of the numerical simulations.

**Figure 3:**
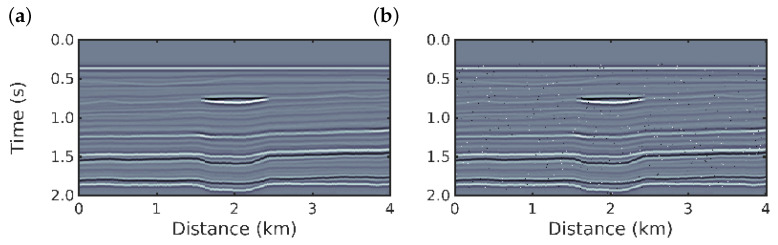
Observed post-stack data: (**a**) noiseless data (first scenario); and (**b**) spiky-noise data (second scenario).

We considered two scenarios: in the first scenario, an ideal case with noiseless data is considered to demonstrate the good working order of the algorithms. Then, in the second scenario, we considered a dataset with spikes, to simulate a non-Gaussian noise. The spikes were added over 1% of the samples (chosen randomly using a uniform distribution) by rescaling the signal amplitudes by a factor of 15β, in which β follows a standard Gaussian distribution. For each scenario, we performed 16 inversions, the first follows the conventional approach, Equation (1) and for the others we used the *q*-PSI with q=0.1,0.3,0.5,…,2.9, Equation (26). The observed data for these two numerical experiments are depicted in Figure 3. We emphasize that the initial model is the same for all numerical experiments.

**Figure 4:**
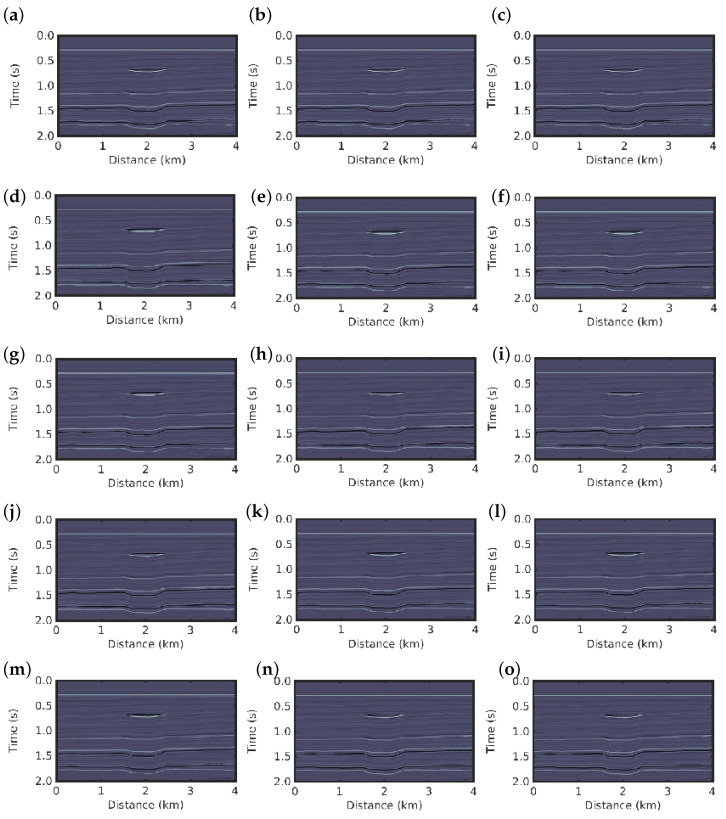
Inversion results for the first scenario: reflectivity models for the *q*-PSI with (**a**) q=0.3, (**b**) q=0.5, (**c**) q=0.7, (**d**) q=0.9, (**e**) conventional PSI, and *q*-PSI with (**f**) q=1.1, (**g**) q=1.3, (**h**) q=1.5, (**i**) q=1.7, (**j**) q=1.9, (**k**) q=2.1, (**l**) q=2.3, (**m**) q=2.5, (**n**) q=2.7, (**o**) q=2.9.

The inversion results for the first scenario using conventional PSI and *q*-PSI with q=0.1,0.3,0.5,…,2.9 are shown in Figure 4. The results for both methods show a good image resolution of the subsurface, we then compare the results with the true reflectivity model (Figure 1b).

**Figure 5:**
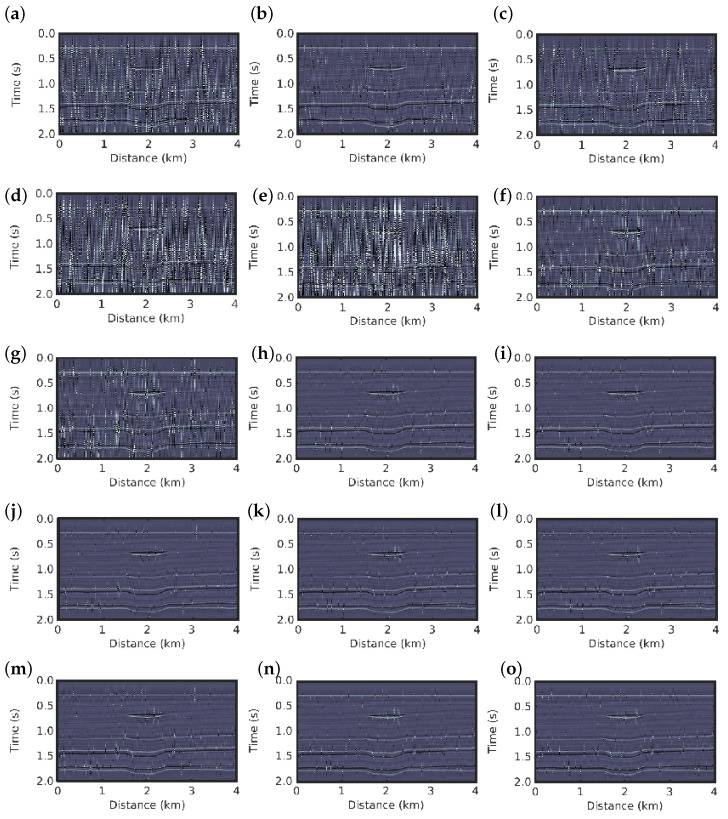
Inversion results for the second scenario: reflectivity models for the *q*-PSI with (**a**) q=0.3, (**b**) q=0.5, (**c**) q=0.7, (**d**) q=0.9, (**e**), conventional PSI, and *q*-PSI with (**f**) q=1.1, (**g**) q=1.3, (**h**) q=1.5, (**i**) q=1.7, (**j**) q=1.9, (**k**) q=2.1, (**l**) q=2.3, (**m**) q=2.5, (**n**) q=2.7, (**o**) q=2.9.

In the second scenario in which non-Gaussian noise is considered, the conventional PSI fails to obtain a good reconstruction of the reflectivity model, as depicted in Figure 5e. In contrast, the *q*-PSI seems to obtain results that are very close to the true reflectivity model as the *q* value increases in the 1<q<3 case; that is, as the deviation from Gaussian behaviour is larger, as depicted in Figure 5f–o for q=1.1,1.3,1.5,1.7,…,2.9, respectively. Although the *q*-PSI results for q≤1.3 show artefacts (see Figure 4a–d,f,g), they are still superior to the conventional PSI result (Figure 4e).

In addition, we compute three statistical measures to quantitatively compare the PSI results with the true reflectivity model: in the first measure, we computed the normalized root-mean-square (NRMS), which is defined as:

(32) $$\mathrm{NRMS}={\left[\frac{\sum_{i=1}^{n}{\left(r_{i}^{true}-r_{i}^{inv}\right)}^{2}}{\sum_{i=1}^{n}{\left(r_{i}^{true}\right)}^{2}}\right]}^{1/2}$$

where $r^{true}$ corresponds to the true reflectivity model and $r^{inv}$ is the inversion result. A NRMS close to 0 means low error. The other two measures are similarity measures: Pearson’s coefficient (R) [37] and the structural similarity (SSIM) index [38]. Pearson’s coefficient measures the linear relationship between the reconstructed model and the true model, while the SSIM is a quality index of similarity between images. Both R and SSIM measures vary between −1 (anti correlation/similarity) to 1 (perfect correlation/similarity). The statistical measures for the two scenarios are summarized in Table 1.

In the analysis of the results presented in Table 1, we observe that the *q*-PSI inversion for the q-value around 2.1 shows the best result with the lowest NRMS and the highest similarity (highest R and SSIM) for all scenarios. However, we emphasize that our proposal with q>1.3 also produces good results, is robust to non-Gaussian noise, and shows low sensitivity to outliers in the dataset, which are represented here by the spikes.

**Figure 6:**
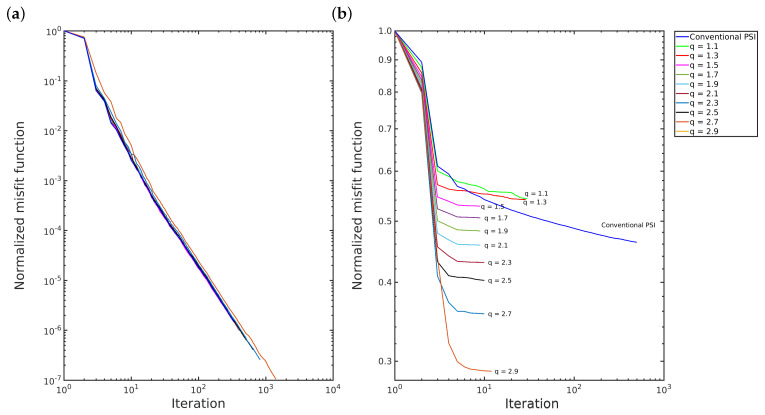
Convergence: (**a**) first scenario; and (**b**) second scenario for 1<q<3 case.

**Figure 7:**
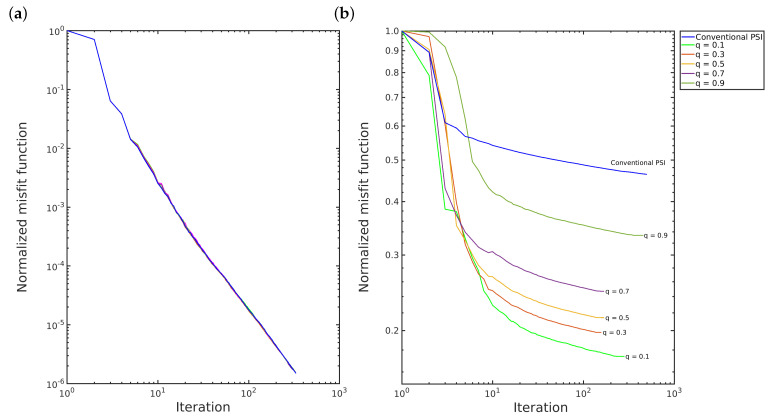
Convergence: (**a**) first scenario; and (**b**) second scenario for 0<q<1 case.

Furthermore, the *q*-PSI requires the same or fewer number of iterations than the conventional PSI to converge, as illustrated in Figure 6. In the first scenario, the convergence of both methods is quite similar, as depicted in Figure 6a and Figure 7a. However, in the second scenario, the *q*-PSI with q>1.3 and q<1 converges more quickly than the others, in which the decay of the convergence curve slopes more as the q-value increases, as depicted in Figure 6b and Figure 7b.

## 5. Conclusions

We investigate the portability of Tsallis nonextensive entropy to compute physical parameters for a geophysical data inverse problem. In this context, we have presented a robust misfit function to mitigate the seismic inversion sensitivity to noise in the reconstruction of subsurface reflectivity models. Given that we employ a PSI method for the geophysical inversion, we refer to our proposal with the abbreviation *q*-PSI—in reference to Tsallis statistics (or *q*-statistics).

To illustrate the stability and robustness of our proposal, we considered two numerical experiments: the first experiment is an ideal noiseless data and in the second experiment we added spikes in 1% of the data simulating a non-Gaussian noise. The numerical experiments demonstrate that the *q*-PSI outperforms the conventional PSI and is a promising tool for seismic exploration, especially for use with non-Gaussian noisy-data. Furthermore, the inclusion of Tsallis statistics in solving the inverse problem under study accelerates the algorithmic convergence of the inversion method, especially for the 1.3<q<3.0 cases.

The choice of the *q*-parameter is an important aspect of our methodology and, after a statistical analysis of the PSI results, we conclude that q-values around 2.1 produce more reliable estimates of the physical parameters. To conclude, the *q*-PSI is a valuable tool in exploration geophysics. Furthermore, we believe that our proposal can be extended to other inverse problems that require robust methods.

## Figures and Tables

**Table 1:** Main statistics of the PSI results.

Strategy	First Scenario				Second Scenario		
	NRMS	R	SSIM		NRMS	R	SSIM
Our proposal (q=0.1)	0.8369	0.8282	0.8277		2.4821	0.4302	0.2756
Our proposal (q=0.3)	0.8366	0.8293	0.8288		2.7015	0.4128	0.2523
Our proposal (q=0.5)	0.8365	0.8287	0.8282		1.3092	0.6187	0.6215
Our proposal (q=0.7)	0.8367	0.8292	0.8286		1.3186	0.6129	0.6152
Our proposal (q=0.9)	0.8369	0.8295	0.8289		4.5209	0.3395	0.1495
**Conventional PSI (q→1.0)**	**0.8373**	**0.8292**	**0.8286**		**6.5366**	**0.3118**	**0.1222**
Our proposal (q=1.1)	0.8370	0.8296	0.8290		2.0517	0.5514	0.4328
Our proposal (q=1.3)	0.8371	0.8293	0.8288		2.1844	0.5362	0.4085
Our proposal (q=1.5)	0.8366	0.8294	0.8288		1.0115	0.7015	0.6934
Our proposal (q=1.7)	0.8374	0.8293	0.8287		1.0057	0.7040	0.6971
Our proposal (q=1.9)	0.8366	0.8289	0.8284		0.9896	0.7083	0.7037
Our proposal (q=2.1)	0.8376	0.8293	0.8287		0.9884	0.7085	0.7041
Our proposal (q=2.3)	0.8371	0.8296	0.8290		0.9966	0.7074	0.7018
Our proposal (q=2.5)	0.8373	0.8290	0.8284		1.0370	0.6951	0.6833
Our proposal (q=2.7)	0.8373	0.8293	0.8287		1.0051	0.7040	0.6971
Our proposal (q=2.9)	0.8376	0.8280	0.8274		1.0178	0.7035	0.6946

## Abbreviations

The following abbreviations are used in this manuscript:

BGS	Boltzmann-Gibbs-Shannon
MEP	Maximum entropy principle
NRMS	Normalized root mean square
PSI	Post-stack inversion
SSIM	Structural similarity
